# The Prognostic Impact of Additional Molecular and Cytogenetic Abnormalities on AML Patients with NPM1- and/or FLT3-ITD Mutations Receiving Intensive Chemotherapy: Real-World Data from the Greek Registry

**DOI:** 10.3390/cancers17040667

**Published:** 2025-02-16

**Authors:** Ioanna Lazana, Maria Papathanassiou, Ioannis Konstantellos, Tatiana Tzenou, Anastasia Kopsaftopoulou, Maria Liga, Vasiliki Violaki, Lydia Kyriazopoulou, Konstantinos Gkirkas, Apostolia Papalexandri, Eleni Plata, Evrydiki Michalis, Theoni Leonidopoulou, Maria Garofalaki, Anastasia Sioni, Irene Tziotziou, Chrysavgi Lalayanni, Dimitrios Kiousiafes, Theodoros P. Vassilakopoulos, Eleni Kapsali, Alexandros Spyridonidis, Ioannis Baltadakis, Maria Angelopoulou, Ioanna Sakellari, Panagiotis Tsirigotis

**Affiliations:** 1 Hematology Division, 2nd Department of Internal Medicine, “ATTIKON” General University Hospital, National and Kapodistrian University of Athens, 11527 Athens, Greece; ilazana@doctors.org.uk (I.L.); konstion4@gmail.com (I.K.); kostas_girkas@yahoo.gr (K.G.); 2 Hematology and Bone Marrow Transplantation Department, Papanikolaou General Hospital, 56403 Thessaloniki, Greece; ppthsmr@gmail.com (M.P.); lila.papalexandri@gmail.com (A.P.); luizana6@gmail.com (C.L.); ioannamarilena@gmail.com (I.S.); 3 Hematology-Lymphoma and Bone Marrow Transplantation Department, Evaggelismos General Hospital, 10676 Athens, Greece; tatianatze@hotmail.com (T.T.); mariakg@yahoo.com (M.G.); tziotzioueirini@gmail.com (I.T.); ibaltadakis@icloud.com (I.B.); 4 Hematology and Bone Marrow Transplantation Department, LAIKON General Hospital, National and Kapodistrian University of Athens, 11527 Athens, Greece; an.kopsaftopoulou@gmail.com (A.K.); eleniplata@gmail.com (E.P.); theopvass@hotmail.com (T.P.V.); mkangelop@med.uoa.gr (M.A.); 5 Hematology and Bone Marrow Transplantation Department, University Hospital of Patras, Rio, 26504 Patras, Greece; marialiga18@yahoo.it (M.L.); spyridonidis@gmail.com (A.S.); 6 Department of Hematology, General Hospital of Chania, 73300 Chania, Greece; vikiviolaki@yahoo.gr; 7 Department of Hematology, University Hospital of Ioannina, 45500 Ioannina, Greece; lkyriazop@me.com (L.K.); elkapsali@gmail.com (E.K.); 8 Department of Hematology, Genimatas General Hospital, 11527 Athens, Greece; eviamich@gmail.com; 9 Department of Hematology, Sismanogleion General Hospital, 15126 Athens, Greece; theonito@yahoo.gr (T.L.); dkousiaphes@gmail.com (D.K.); 10 Department of Hematology, Agios Savvas Cancer Center, 11522 Athens, Greece; anassioni@yahoo.gr

**Keywords:** AML, NPM1, FLT3-ITD, MDS-related mutations, secondary mutations, cytogenetic abnormalities

## Abstract

**Background/Objective:** The prognostic impact of additional cytogenetic aberrations and molecular abnormalities (such as MDS-related mutations, mutations in myeloid genes and the *KRAS/NRAS* mutations) in patients with *NPM1*- and/or *FLT3*-ITD-mutated AML remains elusive. **Methods:** This retrospective, multicentre study of real-world data aimed to investigate the impact of these mutations and cytogenetic abnormalities on the prognosis of patients with *NPM1*- and/or *FLT3*-ITD-mutated AML, treated with intensive chemotherapy. **Results:** In a cohort of 161 patients, the only parameters identified to affect the outcomes (EFS and OS) were the age of the patient, primary refractory disease, the presence of a *NPM1* mutation and the use of allogenic stem cell transplantation (allo-SCT) within the first complete remission. More specifically, ages below the median conferred significantly improved outcomes, whereas primary refractory disease exhibited a negative correlation with the EFS and OS. Subsequent subgroup analysis, stratifying patients into three groups (Group 1: *NPM1*^mutated^/*FLT3*^wt^; Group 2: *NPM1*^mutated^/*FLT3*^mutated^; Group 3: *NPM1*^wt^/*FLT3*^mutated^). revealed that allo-SCT in CR1 improved the outcomes (EFS and OS) in Groups 2 and 3, but had no additional impact in Group 1. **Conclusions:** Age, primary refractory disease and allogenic stem cell transplantation in the first complete response were found to have a prognostic impact on outcomes, Interestingly, no significant association was detected between the poor prognostic cytogenetic abnormalities or the presence of additional mutations in myeloid genes, MDS-related genes or KRAS/NRAS genes and the outcomes in any group of patients.

## Simple Summary

Mutations in the nucleophosmin-1 (*NPM1*) gene and the internal tandem duplications (ITDs) of the fms-related tyrosine kinase 3 (*FLT3*) gene constitute two of the commonest mutations in Acute Myeloid Leukemia (AML), with a significant impact on prognosis. However, the impact of additional abnormalities on outcomes remains elusive. The aim of our retrospective study of real-world data was to investigate whether the presence of additional cytogenetic aberrations and gene mutations have any impact on the outcomes of patients with *NPM1*- and/or *FLT3*-ITD mutated AML treated with intensive chemotherapy. Only age, primary refractory disease and allogenic stem cell transplantation in the first complete response were found to have a prognostic impact on outcomes, whereas the presence of poor prognostic cytogenetic abnormalities or additional mutations had no prognostic significance, which is of particular importance for patient counselling and treatment planning.

## 1. Introduction

The prognostic role of pre-treatment cytogenetic abnormalities and gene mutations in stratifying patients with acute myeloid leukemia (AML) to favourable, intermediate and adverse groups, defining the clinical outcomes, has been established since the first version of the European LeukemiaNet (ELN) [1,2]. Subsequent editions of the ELN recommendations in 2017 and 2022 modified the genetic risk classification to incorporate new mutations and to modify the impact of previously included mutations, either in isolation or in combination with certain cytogenetic abnormalities [3,4].

Two common mutations in AML, with significant impacts on prognosis, include the mutations in the nucleophosmin 1 (*NPM1*) gene and the internal tandem duplications (ITD) of the fms-related tyrosine kinase 3 (*FLT3*) gene [5,6,7,8,9]. *NPM1* mutations have been associated with favourable prognosis in the absence of concomitant *FLT3*-ITD mutations in cytogenetically normal AML [8]. The 2017 ELN recommendations required the determination of low and high allelic ratios for *FLT3*-ITD, considering the *NPM1^mut^*^/^*FLT3*-ITD^low/neg^ status as favourable, regardless of concomitant cytogenetic abnormalities [3,10,11]. However, in the recently updated 2022 ELN recommendations, the *FLT3*-ITD allelic ratio was no longer considered in risk stratification, with AML with *FLT3*-ITD mutation included in the intermediate risk group, irrespective of the presence of *NPM1* mutation [4]. Furthermore, the prognostic significance of genetic alterations was reflected by the incorporation of *NPM1* mutations with adverse-risk cytogenetics into the adverse risk group, based on a meta-analysis correlating *NPM1*-mutated AML with adverse cytogenetic abnormalities with a poor outcome [12]. Finally, beyond the previously included *ASXL1* and *RUNX1* genes, another seven myelodysplasia-related mutations (*BCOR*, *EZH2*, *SF3B1*, *SRSF2*, *STAG2*, *U2AF1*, *ZRSR2*) were placed into the adverse risk group, further emphasizing the need for mutational analysis in all patients, irrespective of their cytogenetic status [4]. Whether the impact of these myelodysplasia-related mutations or other genetic abnormalities have any adverse impact on the otherwise ‘favourable’ *NPM1*-mutated AML remains to be determined. The ELN-2022 prognostic stratification of AML classifies patients with *NPM1*^wt^/*FLT3*-ITD and concurrent adverse risk cytogenetics in the poor risk category, although this assumption is not based on solid data [4]. Another important issue that complicates further the interpretation of data is the potential impact of the type and intensity of treatment on patient outcomes. Indeed, the ELN-2022 prognostic risk stratification was based on data from patients treated intensively and does not apply to patients treated with less intensive approaches such as the combination of azacytidine plus venetoclax. Recently, the ELN-group proposed a genetic risk classification for patients receiving less intense therapies [13]. The presence of certain mutations, such as the *TP53* mutation, and signalling gene mutations (such as the *FLT3*-ITD, *KRAS* and *NRAS* mutations) were related with inferior outcome in this patient group.

The aim of our study was to evaluate the impact of additional gene mutations and cytogenetic abnormalities on the prognosis of AML patients with *NPM1* and/or *FLT3*-ITD mutations, receiving intensive chemotherapy. This is a retrospective, multicentre analysis of real-world data, including patients of all ages treated with intensive chemotherapy.

## 2. Materials and Methods

### 2.1. Patients and Treatment

**Figure 1:**
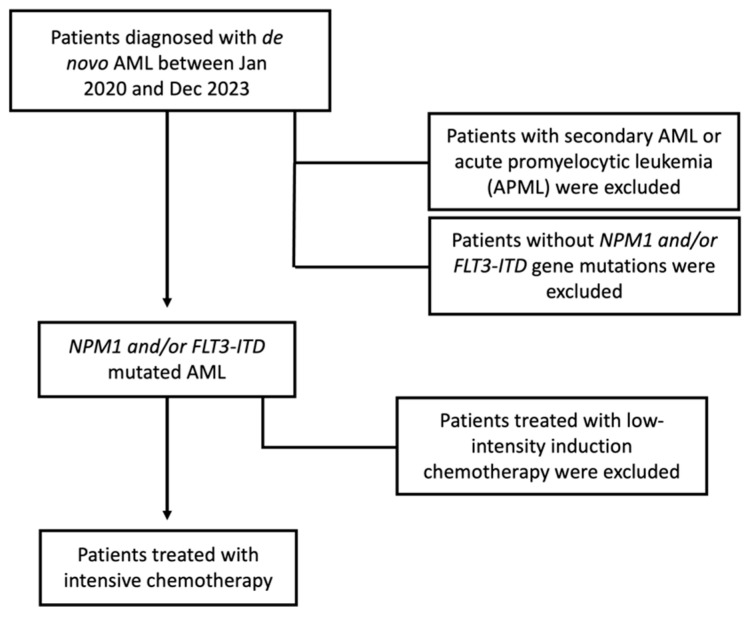
Flow chart of the inclusion and exclusion criteria used for patient selection.

Individual patient data were collected from the Greek AML registry. Patients, aged > 18 years old, with de novo AML, carrying the mutation(s) *NPM1* and/or *FLT3*-ITD, who were treated with intensive chemotherapy between January 2020 and December 2023, were included in the study (Figure 1). The intensive chemotherapy regimen included cytarabine/idarubicin-based induction, followed by consolidation with high- or intermediate-dose chemotherapy. Patients with *FLT3*-ITD mutated AML also received midostaurin as an FLT3 inhibitor. A pre-defined data set was collected, including the following: age at diagnosis, sex, *NPM1*/*FLT3*-ITD mutational status, karyotype, other mutational gene analysis (myelodysplasia gene mutations and *KRAS*/*NRAS*), type and response to induction chemotherapy, events (treatment failure, relapse, death) and date of last contact. Patients with acute promyelocytic leukemia and secondary AML were excluded from the study.

The study protocol was approved by the Institutional Review Board of each participating centre (Primary IRB Approval No. 667/16-11-2022) and was conducted in accordance with the Declaration of Helsinki. Patients gave written informed consent before entry to the study.

### 2.2. Cytogenetic and Molecular Genetic Analysis

Cytogenetic and molecular analysis was performed on the initial diagnostic sample. *NPM1* and *FLT3*-ITD mutation analysis was performed by polymerase chain reaction (PCR) and/or next-generation sequencing (NGS), and the results were used to stratify patients into three groups: (i) patients with *NPM1* mutation (*NPM1^mut^*) and an absence of *FLT3*-ITD (*FLT3*^wt^), (ii) with *NPM1^mut^* and *FLT3*-ITD^mut^ and (iii) with *NPM1*-wild type (*NPM1*^wt^) and *FLT3*-ITD^mut^. The mutational status of the *NPM1*, *FLT3-ITD*, *ASXL1*, *BCOR*, *EZH2*, *RUNX1*, *SF3B1*, *SRSF2*, *STAG2*, *U2AF1*, *ZRSR2*, *KRAS* and *NRAS* genes was determined by NGS in validated laboratories.

Cytogenetic analysis of pre-treatment bone marrow (BM) and/or blood samples was performed in validated central laboratories. Analysis of >20 metaphases from BMs was required to label a specimen as ‘cytogenetically normal’ (CN). Poor/adverse prognosis cytogenetic abnormalities were defined according to the 2022 ELN recommendations.

### 2.3. Clinical Endpoints and Statistical Analysis

Statistical analysis aimed to identify parameters associated with event-free survival (EFS) and overall survival (OS) in patients with AML. EFS was defined as the time from the start of treatment until the occurrence of an event or last follow-up. For the purpose of this analysis, primary refractory disease, relapse after achievement of complete remission (CR), non-achievement of CR/CRi after two cycles of intensive induction chemotherapy, and death from any cause were considered as events. Primary refractory disease was defined as non-achievement of CR/Cri after 2 cycles of induction chemotherapy and was considered an event at the point at which salvage chemotherapy was started. Overall survival (OS) was defined as the time from treatment commencement until the last follow up or death due to any cause.

Categorical variables between groups were compared using Fisher’s two-sided exact test. The Cox proportional hazards regression model was used for multivariate analysis. The following variables were entered in the multivariate analysis model: (1) age at the time of diagnosis (above vs. below the median), (2) sex (male vs. female), (3) presence of abnormal karyotype (yes vs. no), (4) presence of poor risk cytogenetic abnormalities (yes vs. no), (5) presence of mutations in other genes (yes vs. no), (6) presence of mutations in MDS-related genes (yes vs. no), (7) presence of NRAS/KRAS mutations (yes vs. no), (8) primary refractory disease (yes vs. no), and (9) allo-SCT in CR1 (yes vs. no). Multivariate analysis was performed in the whole cohort of patients as well as in Groups 1, 2 and Group 3 separately.

Statistical analysis was performed with the use of easy R and Medcalc statistical software, version 4.1.2.

## 3. Results

### 3.1. Baseline Patient Characteristics and Risk-Group Classification

One hundred and sixty-one patients with new diagnosis of de novo AML were included in the study. Eighty-nine were men and seventy-two were women. The median age at diagnosis was 54 years (range 24–77). The karyotype was normal in 130 patients and abnormal in 31 cases. Of the 31 patients with an abnormal karyotype, 11 belonged to the adverse risk group, according to the 2022 ELN criteria. Mutational analysis was performed in 61 patients, with 41/61 (67%) of patients carrying additional molecular abnormalities on NGS.

Patients were then stratified according to the *NPM1*/*FLT3*-ITD mutational status into three groups. In total, 71 patients (44%) belonged to Group 1 (*NPM1^mut^*/*FLT3*^wt^), 40 patients (25%) belonged to Group 2 (*NPM1^mut^*/*FLT3*-ITD^mut^) and 50 patients (31%) belonged to Group 3 (*NPM1*^wt^/*FLT3*-ITD^mut^). The baseline characteristics of the patients are depicted in Table 1.

Abnormal cytogenetic abnormalities were detected in 9/71, 4/40 and 18/50 patients from Groups 1, 2 and 3, respectively, with a very low number of patients (1/71, 1/40 and 9/50 patients of Groups 1, 2 and 3, respectively) belonging to the adverse risk group. The abnormal and poor prognosis karyotypes were seen significantly more frequent in the group of patients with an *FLT3*-ITD/*NMP1*^wt^ mutation profile (Group 3), as compared with patients in Group 1 and 2 (Table 1). Additional molecular abnormalities were detected in 19/31, 10/12 and 12/18 patients in Groups 1, 2 and 3, respectively, with 6/31, 2/12 and 6/18 carrying MDS-related mutations and 5/31, 1/12 and 0/18 carrying NRAS or KRAS mutations. There was no significant difference in the occurrence of additional molecular abnormalities between the different groups of patients.

Primary refractory disease was significantly more frequently seen in patients of Group 3, as compared to patients of Groups 1 and 2 (*p* = 0.02). More specifically, 9/71 (13%), 7/40 (17.5%), and 15/50 (30%) of patients in Groups 1, 2, and 3, respectively, exhibited primary refractory disease. Accordingly, allogeneic stem cell transplantation (allo-SCT) in the first complete response (CR1) was performed significantly more often in patients of Group 3, as compared to those in other groups, with 27, 24 and 41 patients having undergone allo-SCT in CR1 in Groups 1, 2 and 3, respectively (*p* < 0.001).

### 3.2. Older Age and Primary Reftactory Disease Are the Most Significant Parameters Associated with Decreased Event-Free Survival and Overall Survival

In order to identify potential factors affecting clinical outcomes, a multivariate analysis for EFS and OS was performed for the whole cohort of 161 patients with de novo AML. The only parameters that were found to significantly affect the outcomes were the age of the patient and primary refractory disease. Details are shown in Table 2.

**Figure 2:**
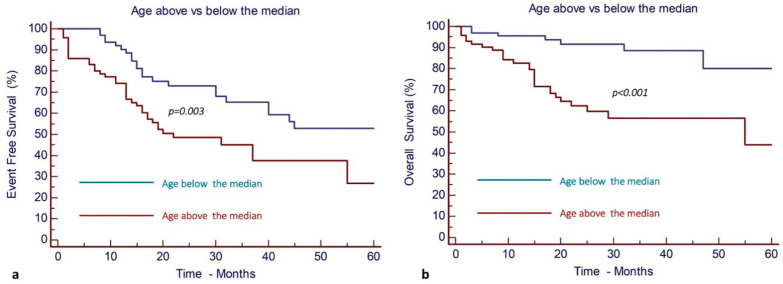
Patient ages above the median result in inferior outcomes compared to ages below the median. (**a**) EFS and (**b**) OS of AML patients.

Patient ages above the median were statistically associated with significantly decreased EFS and OS as compared with ages below the median. After a median follow-up time of 22 months (95% CI, 1925), the EFS and OS of patients aged below the median were 53% (95% CI, 36%67%) and 80% (95% CI, 62–90%), versus 27% (95% CI, 12–44%) and 44% (95% CI, 25–61%) for patients aged above the median, respectively (Figure 2).

**Figure 3:**
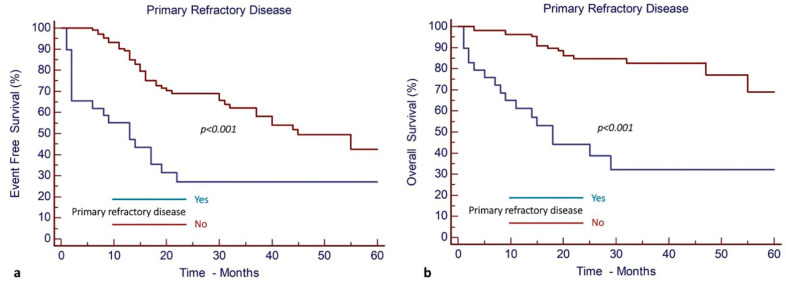
Primary refractory disease has a negative impact on outcomes. (**a**) EFS and (**b**) OS of AML patients.

Similarly, primary refractory disease was associated with significantly decreased EFS and OS. The EFS and OS of patients without primary refractory disease were 42% (95% CI, 28–56%) and 69% (95% CI, 52–80%), respectively, while the EFS and OS were 27% (95% CI, 12–45%) and 32% (95% CI, 14–52%), respectively, for patients with primary refractory disease (Figure 3).

Allo-SCT in CR1 was found to have a significantly positive correlation with the EFS.

More specifically, the EFS was 53% (95% CI, 35–68%) in patients who underwent allo-SCT in CR1, as opposed to 20% (95% CI, 8–36%) for those who did not have allo-SCT in CR1.

The presence of poor-risk cytogenetic abnormalities, the presence of other mutations in myeloid genes, and the presence of mutations in myelodysplasia-related genes and/or mutations in NRAS/KRAS did not seem to have a significant impact on the EFS and OS.

### 3.3. In the Era of FLT3-Inhibitors, Allo-SCT in CR1 Improves the Outcomes of Patients with NPM1^mut^ and FLT3-ITD Mutation (Group 2)

The potential prognostic impact of variable factors (including allo-SCT and primary refractory disease) on outcomes of patients bearing the *NPM1* and/or *FLT3*-ITD mutation(s) (Groups 1–3) was investigated.

No factors were identified to have a significant impact on the patients carrying only the *NPM1*-mutation (Group 1). Furthermore, results confirmed that this group of patients gained no additional benefit in terms of EFS and OS from Allogeneic Stem Cell transplantation (allo-SCT) in CR1.

When multivariate analysis for EFS and OS was performed separately for patients in Group 2 (patients with *NPM1^mut^*/*FLT3*-ITD^mut^), allo-SCT in CR1 and primary refractory disease were the only parameters identified to have a significant impact (Table 3).

**Figure 4:**
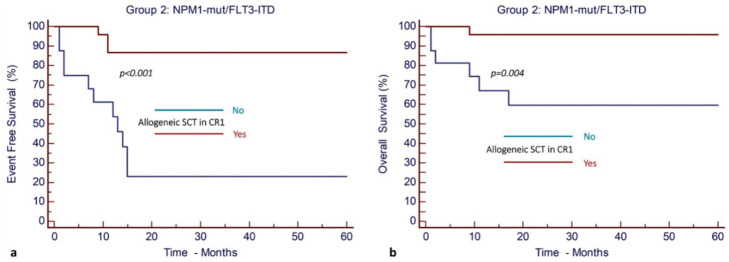
Allogeneic stem cell transplantation in CR1 results in improved outcomes in patients with *NPM1^mut^* and *FLT3*-ITD^mut^ AML (Group 2). (**a**) EFS and (**b**) OS.

Allo-SCT in CR1 was associated with significantly improved EFS and OS. More specifically, after a median follow-up of 23 months (95% CI, 19–46), the EFS and OS were 86% (95% CI, 64–95%) and 95% (95% CI, 74–99%), respectively, for patients who underwent allo-SCT in CR1, as opposed to only 23% (95% CI, 5–47%) and 59% (95% CI, 31–79%), respectively, for the cohort of patients who did not have allo-SCT in CR1 (Figure 4).

**Figure 5:**
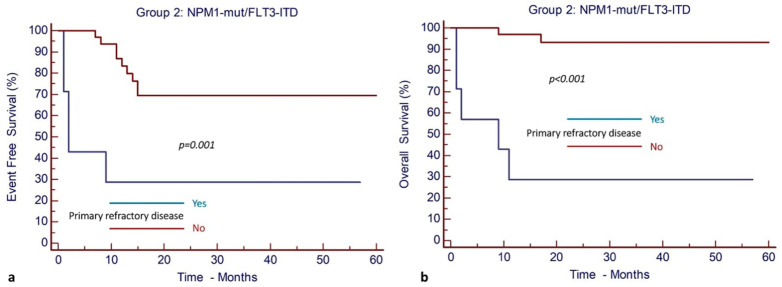
Primary refractory disease has a negative impact on outcomes of patients with *NPM1^mut^* and *FLT3*-ITD^mut^ AML (Group 2). (**a**) EFS and (**b**) OS.

Primary refractory disease was reported to have a significantly negative impact on EFS and OS. More specifically, patients without primary refractory disease exhibited an EFS and OS of 69% (95% CI, 49–82%) and 93% (95% CI, 75–98%), respectively, whereas patients with primary refractory disease had an EFS and OS of 28% (95% CI, 4–61%) and 29% (95% CI, 5–62%), respectively (Figure 5).

There was no association of poor prognostic cytogenetic abnormalities and the presence of other mutations in myeloid genes with the outcome of patients in Group 2

### 3.4. Allogeneic-SCT in CR1 Remains the Most Effective Consolidation for Patients with FLT3-ITD Mutation and Wild-Type NPM1 (Group 3), Even in the Era of FLT3-Inhibitors

In order to identify potential prognostic factors affecting the outcomes of AML patients bearing the *FLT3*-ITD mutation only (Group 3), a multivariate analysis for EFS and OS was performed for the Group 3 of 50 patients (*NPM1*^wt^/*FLT3*-ITD) with AML. The only parameters that remained in the model and that were significantly associated with the outcome were the age of the patient and the performance of allo-SCT in CR1. Details are shown in Table 4.

Patient ages above the median were statistically associated with significantly worse EFS and OS as compared to ages below the median.

**Figure 6:**
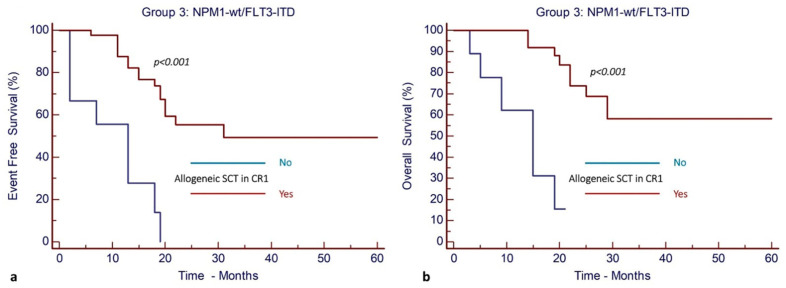
Allogeneic stem cell transplantation in CR1 confers improved outcomes in patients with isolated *FLT3*-ITD gene mutation (Group 3). (**a**) EFS and (**b**) OS.

The performance of allo-SCT in CR1 was associated with significantly improved EFS and OS. After a median follow-up of 19 months, the EFS and OS in the cohort of patients who underwent allo-SCT in CR1 were 49% (95% CI, 29–65%) and 58% (95% CI, 35–75%), respectively. On the contrary, none of the patients who did not receive allo-SCT in CR1 remained free of relapse, resulting in a very low OS of only 18% (Figure 6).

The presence of poor-risk cytogenetic abnormalities, the presence of other mutations in myeloid genes, and the presence of mutations in myelodysplasia-related genes and/or mutations in NRAS/KRAS had no significant effect on the EFS and OS of this group of patients.

## 4. Discussion

Acute myeloid leukemia (AML) is a very heterogeneous disease with variable responses to treatment and outcomes [14]. The impact of cytogenetic aberrations, as well as of molecular abnormalities on the outcomes, is now well established and reflected clearly in the various classifications and risk-stratification guidelines (such as the ELN, ICC and WHO) [3,15,16,17]. Two of the commonest mutations identified in patients with de novo AML, with significant impacts on prognosis, are the mutations in the nucleophosmin 1 (*NPM1*) gene and internal tandem duplications (ITDs) of the fms-related tyrosine kinase 3 (*FLT3*) gene [5,6,7,8,9], with the presence of isolated *NPM1* mutation being associated with favourable prognosis and the presence of *FLT3*-ITD mutation adversely affecting the outcomes. Interestingly, emerging data suggest that the presence of additional cytogenetic abnormalities affect the outcomes of patients with otherwise ‘favourable’ AML (such as the *NPM1*-mutated AML) [12]. This has been reflected on the 2022 updated ELN guidelines, which include *NPM1*-mutated AML with adverse cytogenetic abnormalities in the adverse risk group [4]. Another important parameter that affects the outcome of patients is the type and intensity of treatment. The ELN-2022 guidelines were based on data from patients treated with intensive chemotherapy, and previous studies showed that the ELN-2022 risk stratification is not valid for patients treated with less intensive approaches such as the combination of hypomethylating agents (HMAs) plus venetoclax. Indeed, a recent study showed that the presence of certain mutations (such as the *TP53*, *KRAS/NRAS*, and *FLT3*-ITD) had an adverse impact on the outcome of patients with de novo AML receiving less intensive chemotherapy [13].

The NGS methodology for the detection of mutations in a panel of myeloid genes became widely available recently [18]. Previous studies showed that the median number of driver mutations in patients with AML is 2-3, resulting in a large number of combinations of different mutations present concurrently in the same patient, and raising crucial questions regarding their prognostic impact [19,20]. The ELN-2022 risk stratification proposes that patients with *NPM1^mut^*/*FLT3*^wt^ as well as those with *NPM1^mut^*/*FLT3*-ITD remain in the favourable and intermediate risk category, irrespectively of the presence of additional mutations [4]. However, this assumption is not based on solid data, and the prognostic impact of additional molecular abnormalities (such as the MDS-related mutations) on the outcomes of patients with *NPM1^mut^* and/or *FLT3-ITD* AML is still a matter of debate.

In a multicenter retrospective study of 69 patients with de novo AML treated with intensive chemotherapy, Yi Wang et al. [21] showed that the presence of MDS-related gene mutations does not affect the outcome of patients with *NPM1^mut^*/*FLT3*^wt^ AML. Furthermore, Wright et al. [22], in a multicenter retrospective study of approximately 180 patients with *NPM1*-mut AML, reported that the presence of additional mutations in MDS-related genes did not have a negative impact on treatment outcomes. Similarly to previous findings, a retrospective analysis based on a pooled cohort of 936 *NPM1*-mutated AML patients treated as part of the multicenter trials of the Study Alliance Leukemia (SAL) revealed that MDS-related gene mutations do not have an adverse effect on the treatment outcome of *NPM1^mut^* patients [23].

However, other studies have shown that MDS-related gene mutations have a negative impact on clinical outcome. In a retrospective study based on patients enrolled in GALCB clinical trials, Mrózek et al. showed that myelodysplasia-related gene mutations (MRG-mut) had a negative impact on the outcome of *NPM1^mut^* patients. Based on these data, the authors proposed that *NPM1*-mutated/*FLT3*-ITD-negative patients should be assigned to the intermediate-risk category rather than to the favourable-risk category [24]. Finally, Cocciardi et al. [25] retrospectively evaluated the impact of MRG mutations on the outcome of 568 *NPM1^mut^* AML patients treated with intensive chemotherapy. The authors reported that patients carrying additional MRG mutations, had a higher likelihood of MRD-positive disease at the end on induction and significantly reduced EFS.

The aim of our study was to evaluate the impact of additional gene mutations and cytogenetic abnormalities on the outcomes of AML patients with *NPM1* and/or *FLT3*-ITD mutations receiving intensive chemotherapy in the era of FLT3 inhibitors.

In our study, a cohort of 161 patients with de novo *NPM1*- and/or *FLT3*-ITD-mutated AML were evaluated. The age of the patient and primary refractory disease were the only factors identified to significantly impact the outcomes (EFS and OS) in a negative and positive way, respectively. This is in accordance with previous studies showing the same negative impact of the age [22,26]. However, the presence of adverse risk cytogenetics and mutations in myeloid genes, myelodysplasia-related genes and/or NRAS/KRAS had no impact on the EFS and OS. We then proceeded to stratify patients into three groups according to their *NPM1* and *FLT3*-ITD mutation status and explore the impact of these mutations and cytogenetic aberrations on each group separately. No significant difference was detected in the EFS and OS between Group 1 and Group 2, whereas patients in Group 3 exhibited significantly reduced EFS and a trend toward worse OS, as compared with patients in Groups 1 and 2, suggesting that the presence of *NPM1* mutation overrides the adverse impact of the *FLT3*-ITD mutation. 

It is of particular interest that, in our study, the presence of poor prognostic cytogenetic abnormalities and of additional gene mutations (myeloid genes, MDS-related genes and KRAS/NRAS) had no impact on outcomes (EFS and OS). This is in agreement with a recent retrospective study of 257 patients with *NPM1*-mutated AML showing that MDS-related mutations (therein termed ‘secondary-type’ mutations) have no impact on patient outcomes, suggesting that it is solely the *NPM1* gene mutation driving the disease biology [22]. Similarly, Eckardt et al. [23] observed no significant impact of secondary-type mutations on patient outcomes (CR, EFS and OS) for patients with *NPM1*-mutated AML, despite the fact that patients without secondary-type mutations were younger, with a higher proportion proceeding to allo-SCT. On the contrary, Wang et al. [21] demonstrated a significantly worse PFS (but not OS) for patients with *NPM1*-mutated AML harbouring additional MDS-related gene mutations, although patients with concurrent *FLT3*-ITD mutation exhibited comparable PFS and OS to those with MDS-related gene mutations. This difference in patients with *NPM1* mutations and MDS-related gene mutations between the two studies may be attributed either to the relatively lower number of patients with MDS-related gene mutations in our study (14 vs. 10), or to the potentially different number and/or representation of MDS-related gene mutations in the two cohorts (such as a prevalence of *ASXL1* or *RUNX1* mutations). Similarly, Zhao et al. [27] performed a retrospective analysis of *NPM1*-mutated AML patients with a history of myelodysplastic syndrome (MDS) or of myelodysplastic/myeloproliferative neoplasm (MDS/MPN), (AML-MRC-H). The authors demonstrated that secondary-type mutations were more frequent in patients with AML-MRC-H, compared to those with normal-karyotype *NPM1*-mutated AML (NK-AML), leading to inferior outcomes (OS and EFS). However, in their cohort of patients, there was a prevalence of the secondary-type mutations in *ASXL1* and *U2AF1* in *NPM1^mut^* AML-MRC-H compared to *NPM1^mut^* NK-AML, whereas in our group there was no mutation that stood out, but rather a more equal representation of all mutations. Furthermore, it is hard to isolate the role of the antecedent history of MDS or MDS/MPN when interpreting the role of the secondary-type mutations on outcomes, and any conclusion should be drawn with caution.

The positive impact of allogeneic stem cell transplantation (allo-SCT) in the treatment of AML is beyond what has been established. Our results have confirmed the previously observed lack of added benefits of allo-SCT to patients with isolated *NPM1* gene mutation [28,29] (Group 1). Allo-SCT was found to be associated with significantly improved EFS and OS in patients with *NPM1*-mutated/*FLT3*-ITD mutated (Group 2) and *FLT3*-ITD mutated (Group 3) AML, in agreement with previously reported data demonstrating improved long-term outcomes with allo-SCT for *FLT3*-ITD AML [30,31,32]. However, it has to be noted that the previously reported data referred to patients treated with (intensive) chemotherapy alone, as opposed to our cohort of patients, who were all treated with FLT3 inhibitors in combination with intensive chemotherapy. It is of particular importance that allo-SCT remains a significant contributor to improved outcomes, even in the era of FLT3 inhibitors. This is supported by the recent RATIFY study, where patients with newly diagnosed *FLT3*-ITD-mutated AML received midostaurin in combination with intensive chemotherapy for induction and consolidation. Although allo-SCT was not mandated in the study, the majority of patients underwent allo-SCT, exhibiting a significant survival benefit over those who did not [33]. Nevertheless, there is no evidence so far demonstrating that the use of FLT3 inhibitors provides a survival benefit in *FLT3*-ITD-mutated AML, in the absence of consolidation with Allo-SCT. On the contrary, several retrospective studies have provided evidence for the survival benefit of allo-SCT for patients with FLT3-ITD-mutated AML in the first CR [34]. Finally, it is of interest the fact that there was no association of poor prognostic cytogenetic abnormalities and of additional gene mutations in myeloid genes, MDS-related genes and in KRAS/NRAS with the patient outcomes. Wang et al. [21], studying patients with *NPM1* mutation/*FLT3*-ITD mutation and with *NPM1* mutation/MDS-related mutations, reported no difference in PFS and OS between those who received allo-SCT and those who did not.

## 5. Conclusions

This is a retrospective study analyzing the impact of additional cytogenetic abnormalities and gene mutations (myeloid gene, MDS-related genes and KRAS/NRAS genes) in patients with *NPM1*-mutated and/or *FLT3*-ITD mutated AML, treated with intensive chemotherapy and FLT3 inhibitors, as appropriate. Our results suggest that only age, primary refractory disease and allo-SCT have a prognostic impact on patient outcomes, whereas the additional cytogenetic aberrations and molecular abnormalities have no prognostic significance in this group of patients. Accordingly, patients with *NPM1* gene mutations should be counselled and treated as normally, irrespective of the presence of the aforementioned additional abnormalities.

## Figures and Tables

**Table 1:** Patient characteristics.

	**Group 1**	**Group 2**	**Group 3**	
	*NPM1^mut^*/*FLT3*^wt^	*NPM1^mut^*/*FLT3*-ITD^mut^	*NPM1*^wt^/*FLT3*-ITD^mut^	*p* Value
**No of patients**	71	40	50	
**Male/Female**	38/33	24/16	27/23	*p* = n.s
**Age, years, median, (range)**	52, (26–52)	54, (27–73)	57, (24–77)	*p* = n.s
**Karyotype**				
**Normal**	62/71	36/40	32/50	
Abnormal *	9/71	4/40	18/50	*p* = 0.003
Poor prognosis karyotype *	1/71	1/40	9/50	*p* = 0.001
**Mutation analysis**				
**Not Done**	40/71	28/40	32/50	
**Presence of mutations**	19/31	10/12	12/18	*p* = n.s
**Absence of mutations**	12/31	2/12	6/18	
**MDS-related**	6/31	2/12	6/18	*p* = n.s
**NRAS/KRAS**	5/31	1/12	0/18	*p* = n.s
**DNMT3A-mutations**	8/31	5/12	4/18	*p* = n.s
**Primary Refractory Disease (yes vs. no)**	9/62	7/33	15/35	*p* = 0.02
**Allo-SCT in CR1 (yes/no) ^&^**	27/44	24/16	41/9	*p* < 0.001

* The abnormal and poor prognosis karyotypes were more often present in Group 3 than in Groups 1 and 2. ^&^ Allo-SCT in CR1 was performed more often in Group 3 than in Groups 1 and 2. *Abbreviations:* n.s.: non-significant.

**Table 2:** Multivariate analysis for EFS and OS (all patients, *n* = 161).

Event-Free Survival			
Parameter	Hazard Ratio	95% CI	*p* Value
Age (above vs. below the median)	1.67	0.98–2.88	*p* = 0.06
Primary Refractory Disease (no vs. yes)	0.13	0.06–0.27	*p* < 0.001
Allo-SCT in CR1 * (yes vs. no)	0.17	0.08–0.36	*p* < 0.001
**Overall Survival**			
Age (above vs. below the median)	3.31	1.46–7.50	*p* = 0.004
Primary Refractory Disease (no vs. yes)	0.11	0.05–0.28	*p* < 0.001

* Allo-SCT was entered in the model as a time-dependent covariate.

**Table 3:** Multivariate analysis for EFS and OS (Group 2: *NPM1^mut^*/*FLT3*-ITD).

Event Free Survival			
Parameter	Hazard Ratio	95% CI	*p* Value
* Allo-SCT in CR1 (yes vs. no)	0.05	0.01–0.26	*p* < 0.001
Primary refractory disease (no vs. yes)	0.06	0.01–0.31	*p* < 0.001
**Overall Survival**			
* Allo-SCT in CR1 (yes vs. no)	0.07	0.01–0.65	*p* = 0.019
Primary refractory disease (no vs. yes)	0.04	0.01–0.25	*p* < 0.001

* Allo-SCT was entered in the model as a time-dependent covariate.

**Table 4:** Multivariate analysis for EFS and OS (Group 3: *NPM1*^wt^/*FLT3*-ITD^mut^, n = 50).

Event-Free Survival			
Parameter	Hazard Ratio	95% CI	*p* Value
Age (above vs. below the median)	5.67	1.54–20.8	*p* = 0.008
Allo-SCT in CR1 * (yes vs. no)	0.24	0.09–0.63	*p* = 0.003
**Overall Survival**			
Age (above vs. below the median)	14.4	1.78–116.7	*p* = 0.01
Allo-SCT in CR1 * (yes vs. no)	0.16	0.05–0.567	*p* = 0.003

* Allo-SCT was entered in the model as a time-dependent covariate.

## Data Availability

The data presented in this study are available on request from the corresponding author. The data are part of the Greek AML Registry and the owner is the Hellenic Society of Hematology. Therefore, data are available upon request and after the permission of the Hellenic Society of Hematology is obtained.
